# PARP1 contributes to neuropathic pain via GPX4-dependent sensory neuron ferroptosis in nerve-injured mice

**DOI:** 10.1038/s41420-026-03307-4

**Published:** 2026-08-15

**Authors:** Yihao Guo, Lijin Huang, Yinhao Chen, Yilu Li, Qinming Liao, Jian Liu, Wei Zhao, Jiajun Zhu, Jun Chen, Zhongjie Liu

**Affiliations:** 1 Department of Anesthesia, Zhujiang Hospital, Southern Medical University, Guangzhou, China; 2 Department of Anesthesia, The People’s Hospital of Gaoming District of Foshan City, Foshan, China; 3 Department of Neurosurgery, Guangdong Provincial Hospital of Chinese Medicine (Guangdong Provincial Academy of Chinese Medical Sciences), Guangzhou, China; 4 Department of Neurosurgery, The Third Affiliated Hospital of Southern Medical University, Guangzhou, China; 5 The Second Clinical College of Guangzhou University of Chinese Medicine, Guangzhou, China; 6 Department of Anesthesia, Zhongshan Hospital of Traditional Chinese Medicine Affiliated to Guangzhou University of Chinese Medicine, Zhongshan, China; 7 Department of Neurosurgery, Zhongshan Hospital of Traditional Chinese Medicine Affiliated to Guangzhou University of Chinese Medicine, Zhongshan, China; 8 Department of Anesthesia, Affiliated Shenzhen Children’s Hospital of Shantou University Medical College, Shenzhen, China

**Keywords:** Peripheral nervous system, Cell death

## Abstract

Neuropathic pain is associated with sensory neuron injury, and ferroptosis has been implicated in neuronal damage and death. However, the regulatory mechanism of ferroptosis in neuropathic pain remains unclear. This study aimed to investigate poly-(ADP-ribose) polymerase 1 (PARP1) in regulating sensory neuron ferroptosis and to assess its potential therapeutic role in neuropathic pain. A chronic constriction nerve injury (CCI) mouse model was established to examine how ferroptosis in sensory neurons contributes to CCI-induced neuropathic pain. We analyzed the expression of ferroptosis-related key gene (GPX4, FPN1, FSP1, TFR1, DMT1) and ferroptosis in dorsal root ganglion (DRG) neurons, along with PARP1 activity. Blocking PARP1 activity with PJ34 or olaparib, or conditionally knocking out neuronal PARP1, was performed to assess effects on GPX4 expression, ferroptosis, and neuropathic pain behavior. Nerve injury downregulated GPX4 expression and enhanced ferroptosis in DRG neurons, accompanied by increased PARP1 activity. Using sensory neuron-specific PARP1 conditional knockout mice, we demonstrated that neuronal PARP1 deletion upregulated GPX4 expression, reduced ferroptosis, and alleviated neuropathic pain-like behaviors. Blocking GPX4 activity induced pain sensitization in normal mice. Inhibition of ferroptosis or administration of PARP1 inhibitors significantly alleviated pain-like behaviors in CCI mice. Following nerve injury, PARP1 overactivation drives neuropathic pain by suppressing GPX4 and promoting ferroptosis in DRG neurons. Conditional knockout or pharmacological inhibition of PARP1 reversed these effects and alleviated pain. Thus, the PARP1-GPX4 axis represents a promising therapeutic target.

## Introduction

Neuropathic pain is a global health concern marked by allodynia, hyperalgesia, and spontaneous pain in the absence of external stimuli [1, 2]. Approximately 7–10% of the global population is affected by this condition [3, 4]. Conventional treatments often fail to adequately relieve symptoms because the underlying mechanisms of neuropathic pain remain incompletely understood.

Extensive research has identified nerve injury and inflammatory responses as major contributors to the pathogenesis of neuropathic pain [5, 6]. Recent evidence has revealed that nerve injury can induce deoxyribonucleic acid (DNA) damage in sensory neurons, leading to neuropathic pain-like behavior [7]. The poly(ADP-ribose) polymerase (PARP) family, a group of nuclear enzymes, regulates gene expression and DNA repair through poly(ADP-ribosyl)ation and transcriptional activation [8, 9]. Among these, PARP1 is the principal executor of DNA damage repair. Upon activation, PARP1 binds to DNA strand breaks, forms homodimers, and catalyzes the cleavage of NAD^+^ into nicotinamide and ADP-ribose to facilitate base excision repair [10, 11]. Notably, PARP1 activation depletes NAD^+^ and ATP, inhibits glycolysis and mitochondrial energy metabolism, leading to cellular dysfunction and death [12, 13]. Moderate regulation of PARP1 activity has shown therapeutic promise in cancer treatment [14, 15]. Meanwhile, animal model studies indicated that PARP1 inhibition alleviates neuropathic pain-like behavior among rats [16]. However, the mechanism by which PARP1 regulates sensory neuron death remains unclear.

Ferroptosis is a distinct form of non-apoptotic cell death characterized by iron overload and reactive oxygen species-dependent lipid peroxidation [17]. Imbalances in iron homeostasis and lipid oxidation are central to this process [18]. Abnormal expression of iron transport proteins such as ferroportin 1 (FPN1), transferrin receptor protein 1 (TFR1), and divalent metal transporter 1 (DMT1) disrupts intracellular iron balance and can trigger ferroptosis [19–21]. Two critical regulators of ferroptosis have been identified: Glutathione peroxidase 4 (GPX4) and ferroptosis suppressor protein 1 (FSP1) [22, 23]. When intracellular glutathione (GSH) levels are depleted, GPX4 activity declines, and divalent iron catalyzes excessive lipid formation through the Fenton reaction. The resulting lipid oxides cannot be neutralized through GPX4-mediated GSH reduction, leading to oxidative damage of membrane lipids, loss of membrane integrity, and ultimately ferroptotic cell death [24]. Conversely, FSP1 inhibits ferroptosis independently of GPX4 by reducing coenzyme Q10 and vitamin K, thereby eliminating lipid peroxides [25]. Ferroptosis inhibition has been demonstrated to alleviate multiple pathological conditions such as cancer, neurodegenerative disorders, and neuropathic pain [26, 27]. However, the specific molecular mechanisms underlying ferroptosis in these processes require further investigation. Selenium can enhance GPX4 expression during ferroptosis and protect neurons by activating specificity protein 1 (SP1), which plays an important role in neuronal survival after stroke [28]. Moreover, PARP1 can suppress gene expression through poly(ADP-ribosyl)ation of related transcription factors [29], and its interaction with SP1 is known to influence gene transcription [30]. Whether PARP1 mediates GPX4-dependent neuronal ferroptosis through poly(ADP-ribosyl)ation of SP1 in neuropathic pain remains to be elucidated.

In this study, a chronic constriction injury (CCI) model was established in PARP1 conditional knockout mice and used to investigate the role of GPX4-dependent ferroptosis in dorsal root ganglion (DRG) neurons. The findings demonstrated that GPX4-dependent ferroptosis contributes to the pathogenesis of neuropathic pain, and PARP1 represents a potential therapeutic target for regulating this process.

## Results

### PARP1 inhibition alleviates pain hyperalgesia in CCI mice

**Fig. 1:**
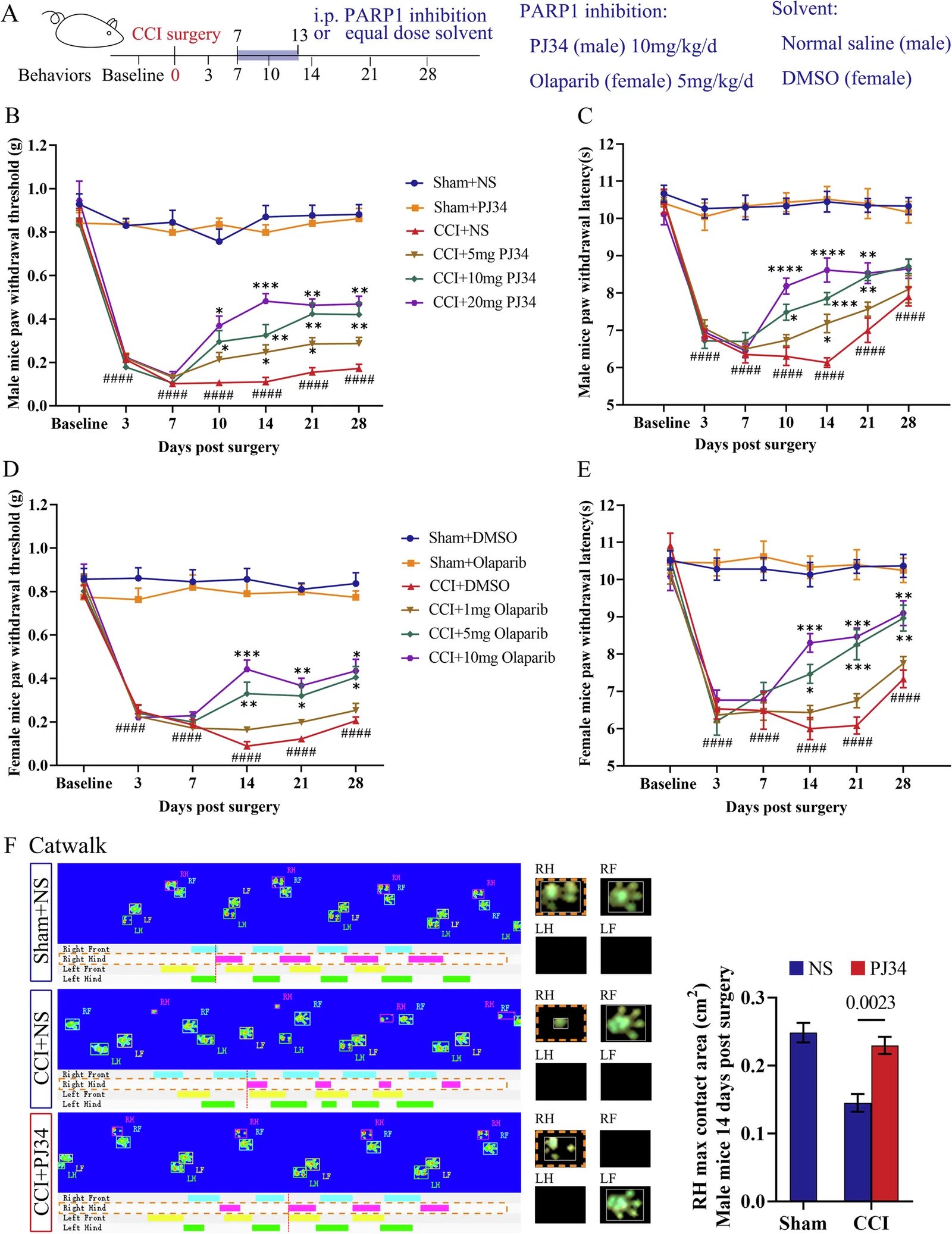
PARP1 inhibition alleviates pain hypersensitivity in CCI mice. **A** Schematic of the experimental design, including PARP1 inhibitor dosage, intervention timeline, and methodology. Starting on postoperative day 7, mice received daily intraperitoneal injections of PARP inhibitors for 7 days (Sham group: 100 μl saline; CCI group: males: PJ34, 10 mg/kg/day,100 μl; females: olaparib, 5 mg/kg/day, 100 μl). Time course of mechanical (**B**) and thermal pain threshold (**C**) in male CCI mice after PJ34(5, 10, or 20 mg/kg). Time course of mechanical (**D**) and thermal pain threshold (**E**) in female CCI mice after olaparib (1, 5, or 10 mg/kg). **F** Catwalk analysis revealed that PJ34 treatment increased the maximum paw contact area in male CCI mice on postoperative day 14. Data are expressed as mean ± SEM (*n* = 6). * vs CCI + NS or CCI + DMSO, *P < 0.05, **P < 0.01, ***P < 0.001; # vs Sham+NS or Sham+DMSO, #*P* < 0.05, ##*P* < 0.01, ###*P* < 0.001, ####*P* < 0.0001; CCI chronic constriction injury, NS normal saline, PJ34 and olaparib PARP1 inhibitions.

A CCI model was established in C57/BL6N mice, with behavioral assessment time points and PARP1 inhibitor intervention were outlined (Fig. 1A). Baseline motor function and pain thresholds were comparable across all groups. Mice developed significant hyperalgesia following CCI surgery. To determine the optimal PARP1 inhibitor dose, we conducted dose-response experiments in CCI mice using PJ34 (5, 10, or 20 mg/kg) and olaparib (1, 5, or 10 mg/kg). Both inhibitors reduced pain hypersensitivity in a dose-dependent manner. The low dose of PJ34 (5 mg/kg) produced only transient and partial effects, while the middle (10 mg/kg) and high (20 mg/kg) doses consistently improved pain thresholds from day 10 onward, with comparable efficacy at later time points. Similarly, olaparib at 5 and 10 mg/kg showed sustained analgesic effects, whereas the 1 mg/kg dose was minimally effective. The middle and high doses of both drugs exhibited comparable efficacy at later time points, indicating that the middle dose was sufficient to achieve maximum effect. Based on these results, the middle doses (PJ34 at 10 mg/kg; olaparib at 5 mg/kg) were selected for subsequent experiments. Using these selected doses, we next assessed the therapeutic effects of PARP1 inhibitors. Administered on postoperative day 7, PJ34 significantly improved both mechanical and thermal pain thresholds in male CCI mice (Fig. 1B, C). Parallel experiments were performed in female mice using olaparib, a clinically approved PARP1 inhibitor. Similar to males, female mice exhibited reduced hyperalgesia after olaparib treatment (Fig. 1D, E). Catwalk gait analysis in male mice further confirmed that PJ34 reversed the reduction in paw contact area typically observed after CCI surgery (Fig. 1F). These results suggest that PARP1 inhibition alleviates hyperalgesia through modulation of DRG neurons.

### PARP1 inhibition reduces ferroptosis in DRG neurons

**Fig. 2:**
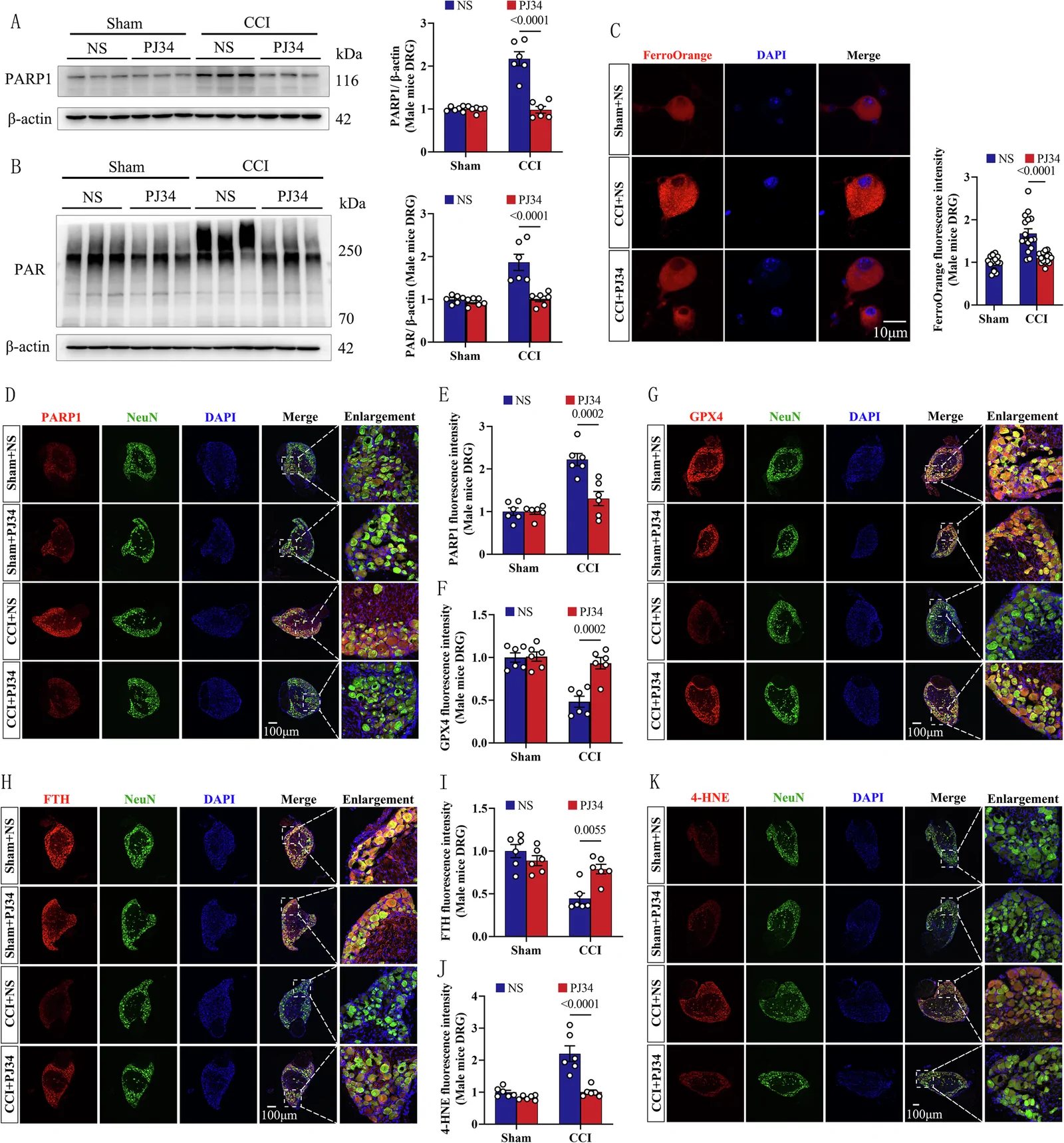
PARP1 inhibition reduces ferroptosis in DRG neurons. Western blot detection and quantification of PARP1 expression in DRG neurons of male (**A**) and female (**B**) mice after CCI surgery. **C** Ferrous iron levels in purified DRG neurons after PARP1 inhibition. FerroOrange is shown in red; DAPI is shown in blue. *n* = 16. Scale bar = 10 μm. Intraperitoneal injection: NS and DMSO: 100 μl; PJ34, 10 mg/kg/d in 100 μl NS. **D**, **E** Immunofluorescence analysis and quantification of PARP1 expression in DRG neurons post-PARP1 inhibition. PARP1 is shown in red; NeuN is shown in green; DAPI is shown in blue. Scale bar = 100 μm. **F**, **G** Immunofluorescence analysis and quantification of GPX4 expression in DRG neurons post-PARP1 inhibition. GPX4 is shown in red; NeuN is shown in green; DAPI is is shown blue. Scale bar = 100 μm. **H**, **I** Immunofluorescence analysis and quantification of FTH expression in DRG neurons post-PARP1 inhibition. FTH is shown in red; NeuN is shown in green; DAPI is shown in blue. Scale bar = 100 μm. **J**, **K** Immunofluorescence analysis and quantification of 4-HNE expression in DRG neurons post-PARP1 inhibition. 4-HNE is shown in red; NeuN is shown in green; DAPI is shown in blue. Scale bar = 100 μm. Data are expressed as mean ± SEM (*n* = 6). FTH ferritin heavy chain, 4-HNE 4-hydroxynonenal.

PARP1 protein expression and fluorescence localization were measured in DRG neurons. The results showed that PARP1 expression and its product PAR were significantly upregulated in DRG neurons, and these levels decreased following intervention with the PARP1 inhibitor (Fig. 2A, B, D, E). To examine iron accumulation, DRG neurons were isolated, purified, and cultured, then stained with FerroOrange to detect intracellular Fe^2+^. A significant increase in Fe^2+^ accumulation was observed in CCI mice, which was substantially attenuated by PJ34 treatment (Fig. 2C). To assess ferroptosis in DRG neurons, the levels of GPX4, ferritin heavy chain (FTH), and 4-hydroxyenal (4-HNE) were measured. Compared with normal mice, CCI mice exhibited reduced GPX4 and FTH expression and elevated 4-HNE levels. In particular, PJ34 treatment reversed these changes, upregulating GPX4 and FTH expression (Fig. 2F–I) while suppressing 4-HNE expression (Fig. 2J, K). These results indicate that PARP1 inhibition alleviates neuropathic pain by mitigating ferroptosis in DRG neurons through regulation of GPX4, ferritin, and iron accumulation.

### Conditional knockout of PARP1 in neurons alleviates pain hypersensitivity in CCI mice

**Fig. 3:**
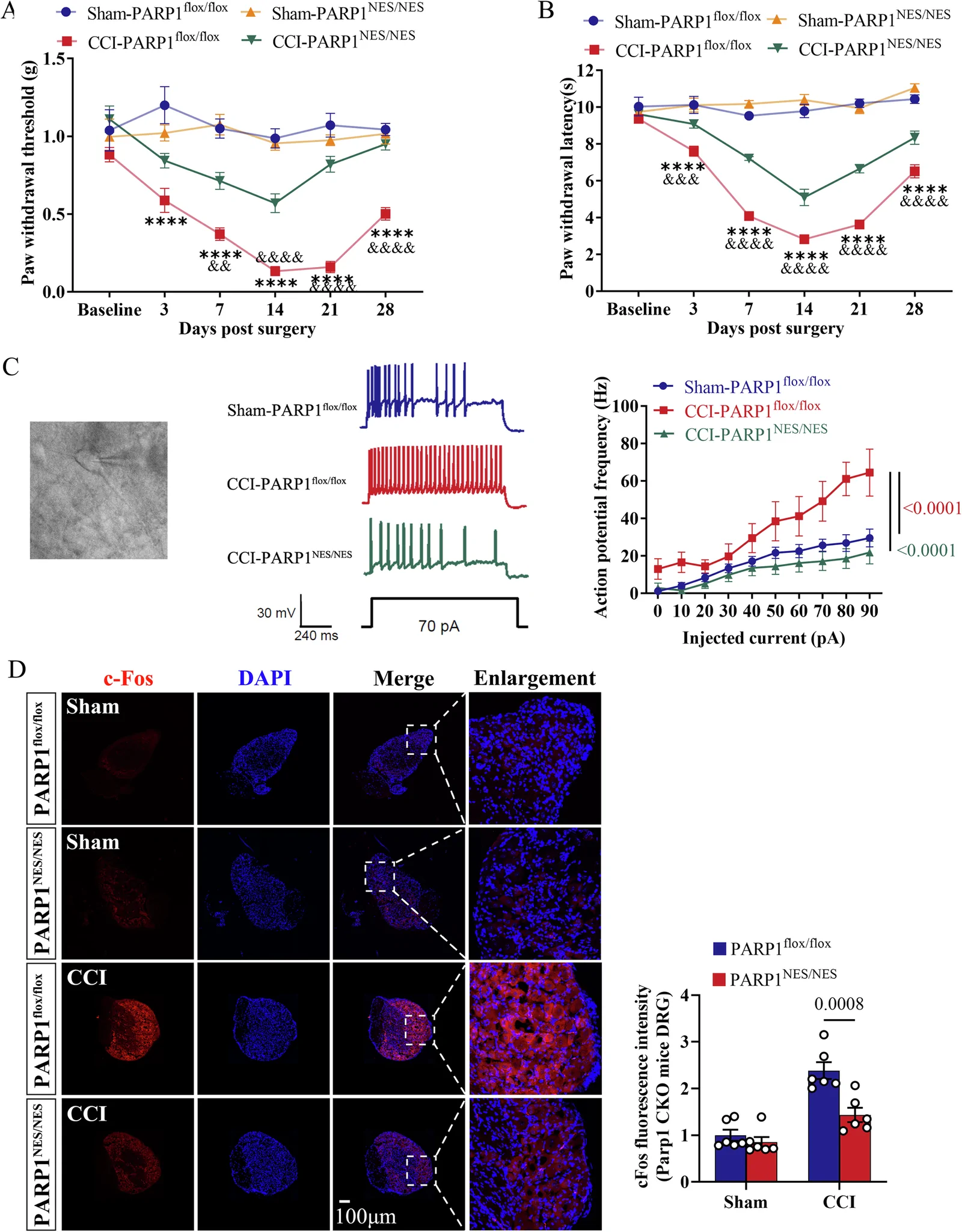
Conditional knockout of PARP1 in neurons alleviates pain hypersensitivity in CCI mice. Time course of mechanical (**A**) and thermal (**B**) pain thresholds in knockout mice post-CCI. **C** Patch-clamp recordings of action potential changes in DRG neurons post-knockout, including the diagram of tissue-contact patch clamp electrodes, responses to current injections (70 pA), and comparison of action potentials frequency among Sham-*PARP1*^flox/flox^, CCI-*PARP1*^flox/flox^ and CCI- *PARP1*^NES/NES^. **D** Immunofluorescence staining and quantification of c-Fos in DRG neurons of knockout mice. c-Fos is shown in red; DAPI is shown in blue. Scale bar = 100 μm. Data are expressed as mean ± SEM (*n* = 6). * vs Sham-*PARP1*^flox/flox^, ns *P* > 0.05, *P < 0.05, **P < 0.01, ***P < 0.001, ****P < 0.0001; & vs CCI-*PARP1*^NES/NES^, *P* < 0.01, *P* < 0.0001.

To investigate the role of PARP1 in DRG neurons, a mouse line with sensory neuron-specific conditional knockout of PARP1 (*PARP1*^NES/NES^) was generated. Successful generation was confirmed by genotyping (Supplementary Fig. 1A), and sensory neuron-specific deletion was validated through qPCR and Western blot analysis across multiple tissues (Supplementary Fig. 1B–E), demonstrating selective PARP1 downregulation in the DRG but not in the heart, liver, or lung. This strain exhibited normal motor function, pain threshold, and perception, indicating that neuronal PARP1 deletion does not impair baseline physiological function. Following CCI surgery, behavioral tests showed that *PARP1*^NES/NES^ mice displayed significantly reduced pain hypersensitivity and faster recovery compared to controls (Fig. 3A, B). Recordings of DRG neuron excitability revealed an abnormal increase in firing frequency in CCI-*PARP1*^flox/flox^ mice, whereas this change was absent in CCI-*PARP1*^NES/NES^ mice (Fig. 3C). Additionally, c-Fos expression, a marker of neuronal activation [31], was elevated in DRG neurons of CCI-*PARP1*^flox/flox^ mice but diminished in CCI-*PARP1*^NES/NES^ mice (Fig. 3D).

### PARP1 mediates GPX4-dependent ferroptosis in DRG neurons of CCI mice

**Fig. 4:**
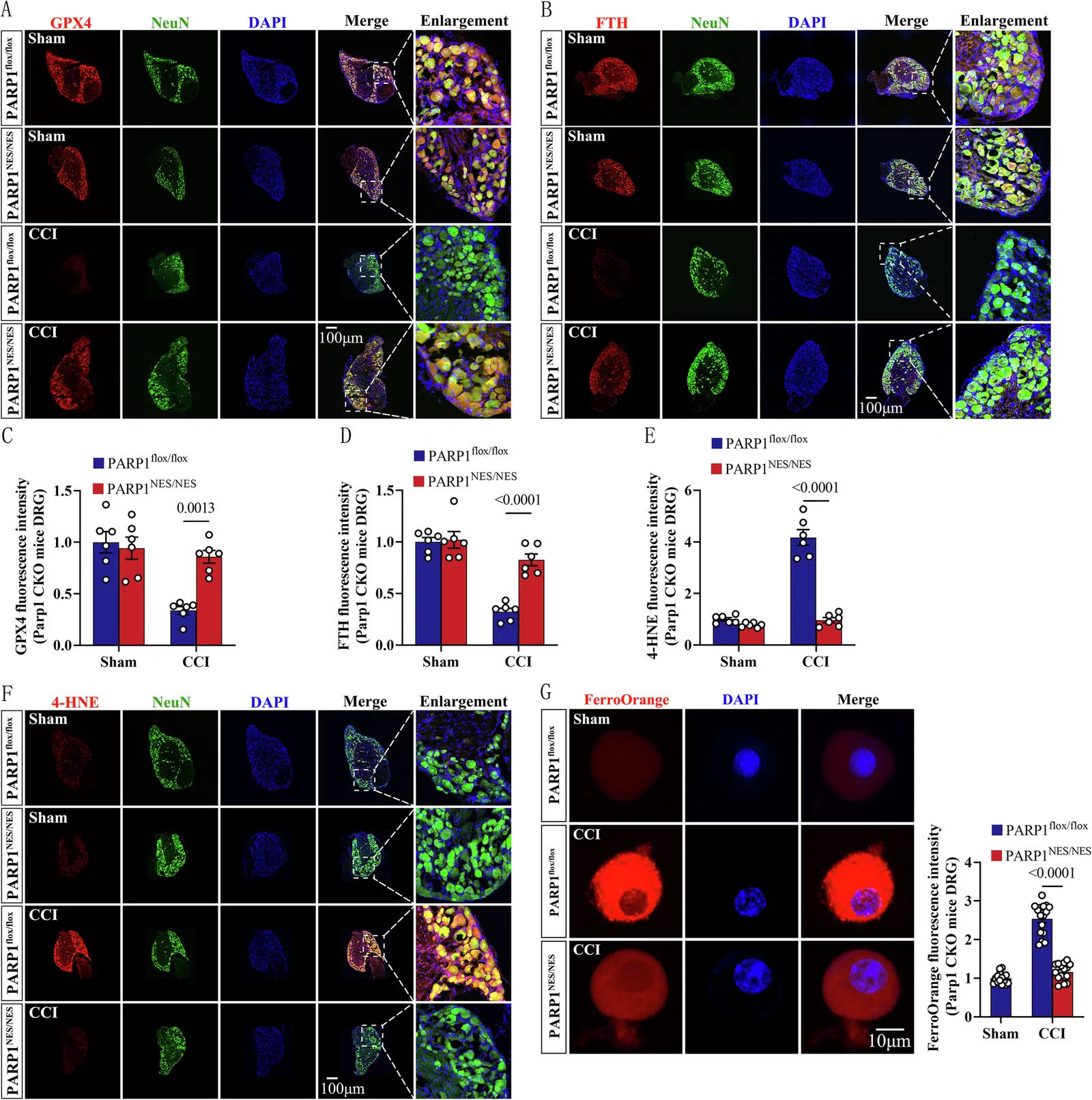
PARP1 mediates GPX4-dependent ferroptosis in DRG neurons of CCI mice. **A**, **C** Immunofluorescence analysis and quantification of GPX4 in DRG neurons post-knockout. GPX4 is shown in red; NeuN is shown in green; DAPI is shown in blue. *n* = 6. Scale bar = 100 μm. **B**, **D** Immunofluorescence analysis and quantification of FTH in DRG neurons post-knockout. FTH is shown in red; NeuN is shown in green; DAPI is shown in blue. *n* = 6. Scale bar = 100 μm. **E**, **F** Immunofluorescence analysis and quantification of 4-HNE in DRG neurons post-knockout. 4-HNE is shown in red; NeuN is shown in green; DAPI is shown in blue. *n* = 6. Scale bar = 100 μm. **G** Ferrous iron levels in cultured DRG neurons from conditional knockout mice. FerroOrange is shown in red; DAPI is shown in blue. *n* = 16. Scale bar = 10 μm. Data are expressed as mean ± SEM.

**Fig. 5:**
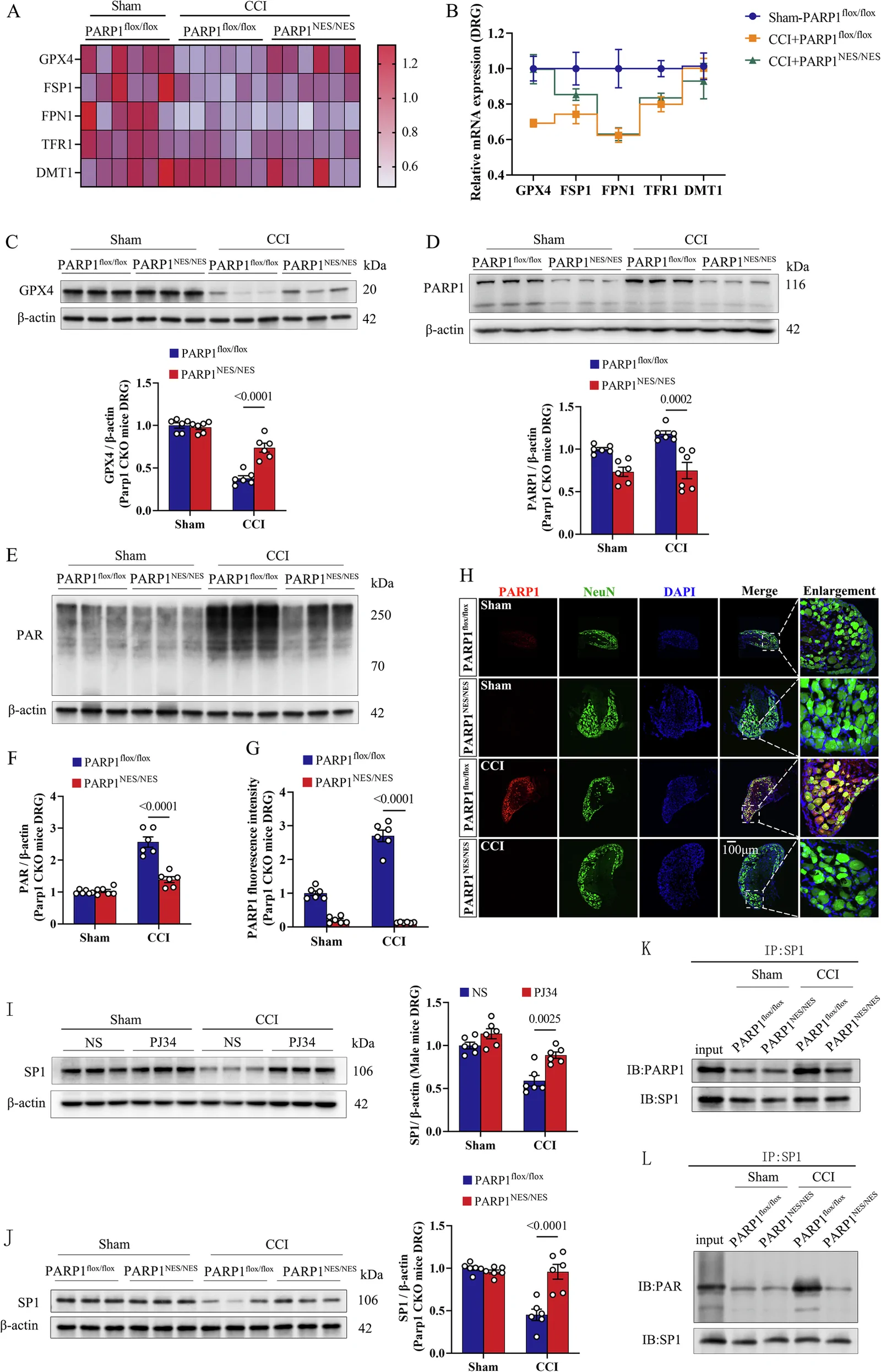
PARP1 interacts with SP1 to mediate GPX4-dependent ferroptosis in DRG neurons of CCI mice. **A**, **B** RT-qPCR analysis of ferroptosis-related genes mRNA levels in DRG neurons of Parp1 knockout mice post-CCI, measured by RT‑qPCR. **C**–**F** Western blot detection and quantification of GPX4, PARP1, and PAR expression in DRG neurons of Parp1 knockout mice post-CCI. **G**, **H** Immunofluorescence staining and quantification of PARP1 in DRG neurons of knockout mice. PARP1 is shown in red; NeuN is shown in green; DAPI is shown in blue. Scale bar = 100 μm. **I** Western blot detection and quantification of SP1 expression in DRG neurons of male mice after CCI surgery. **J** Western blot detection and quantification of SP1 expression in DRG neurons of Parp1 knockout mice post-CCI. **K**, **L** Co-immunoprecipitation of PARP1 and PAR with SP1 in DRG neurons after Parp1 conditional knockout. Data are expressed as mean ± SEM (*n* = 6).

GPX4 and FTH expressions significantly decreased in *PARP1*^flox/flox^ mice after CCI surgery, whereas the decline was less pronounced in *PARP1*^NES/NES^ mice (Fig. 4A–D). 4-HNE expression increased substantially in *PARP1*^flox/flox^ mice after CCI surgery but was reduced in *PARP1*^NES/NES^ mice (Fig. 4E, F). Fe^2+^ accumulation, associated with ferroptosis, was also significantly reduced in *PARP1*^NES/NES^ mice (Fig. 4G), suggesting that PARP1 mediates GPX4-dependent ferroptosis in DRG neuron of CCI mice. To clarify the ferroptosis-related molecular pathway in DRG neurons, we examined the expression of five key ferroptosis-related genes (GPX4, FSP1, FPN1, TFR1, and DMT1) based on previous research in CCI mice [32]. GPX4 levels were markedly lower in CCI-*PARP1*^flox/flox^ mice than in CCI-*PARP1*^NES/NES^ mice, whose expression remained relatively stable (Fig. 5A, B). Accordingly, GPX4 protein levels were significantly downregulated in CCI-*PARP1*^flox/flox^ mice compared to CCI-*PARP1*^NES/NES^ mice (Fig. 5C). Meanwhile, PARP1 and its product PAR were upregulated in *PARP1*^flox/flox^ mice after CCI surgery, but not in PARP1 knockout mice (Fig. 5D–H). SP1 expression in DRG neurons from PJ34-treated mice and *PARP1*^NES/NES^ mice revealed a similar pattern of downregulation following CCI (Fig. 5I, J). Consistent with previous reports that PARP1 mediates poly(ADP‑ribosyl)ation of SP1 [29, 30], co-immunoprecipitation (Co-IP) revealed enhanced PARP1-SP1 interaction in CCI-*PARP1*^flox/flox^ mice (Fig. 5K, L). Collectively, these results indicate that PARP1 interacts with SP1 to drive GPX4-dependent ferroptosis in DRG neurons of CCI mice.

### Induction of GPX4-dependent ferroptosis in DRG neurons drives neuropathic pain in CCI mice

**Fig. 6:**
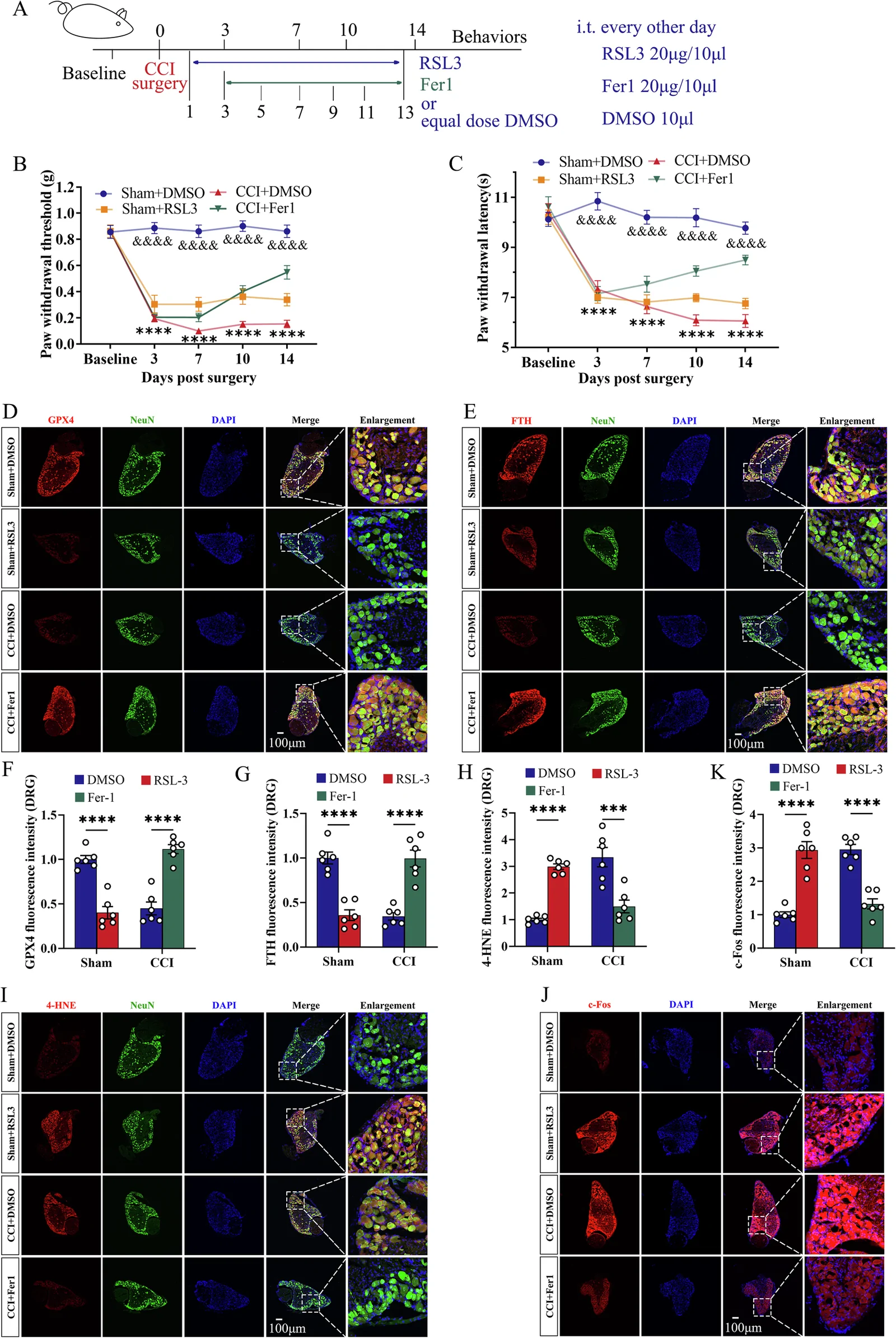
Induction of GPX4-dependent ferroptosis in DRG neurons drives neuropathic pain. **A** Experimental design schematic showing RSL3 and Fer-1 dosages and intervention timeline. Intrathecal injections (every other day): DMSO (10 μl), RSL3 (20 μg/10 μl), Fer-1 (20 μg/10 μl). Time course of mechanical pain threshold (**B**) and thermal (**C**) pain threshold changes in male CCI mice treated with RSL3 or Fer-1. **D**, **F** Immunofluorescence staining and quantification of GPX4 in DRG neurons of male CCI mice treated with RSL3 or Fer-1. GPX4 is shown in red; NeuN is shown in green; DAPI is shown in blue. Scale bar = 100 μm. **E**, **G** Immunofluorescence staining and quantification of FTH in DRG neurons under the same conditions. FTH is shown in red; NeuN is shown in green; DAPI is shown in blue. Scale bar = 100 μm. **H**, **I** Immunofluorescence staining and quantification of 4-HNE in DRG neurons. 4-HNE is shown in red; NeuN is shown in green; DAPI is shown in blue. Scale bar = 100 μm. **J**, **K** Immunofluorescence staining and quantification of c-Fos in DRG neurons. c-Fos is shown in red; DAPI is shown in blue. Scale bar = 100 μm. Data are expressed as mean ± SEM (*n* = 6). * vs Sham+DMSO, ****P < 0.0001. & vs Sham+RSL3, *P* < 0.0001. RSL3: ferroptosis activator; Fer-1: Ferrostatin-1, ferroptosis inhibitor.

To further validate that GPX4-dependent ferroptosis is sufficient to drive neuropathic pain, intrathecal injections of RSL3 (a GPX4 inhibitor) or Fer-1 (a ferroptosis inhibitor) were administered to both sham and CCI mice (Fig. 6A). RSL3 treatment significantly reduced mechanical and thermal pain thresholds, inducing hypersensitivity symptoms similar to those observed in CCI mice. Conversely, Fer-1 administration alleviated pain hypersensitivity in CCI mice (Fig. 6B, C). Immunofluorescence revealed that RSL3 induced ferroptosis in DRG neurons, with decreased GPX4 and FTH expression following RSL3 treatment and after CCI surgery (Fig. 6D–G), suggesting disrupted iron homeostasis in DRG neurons. The ferroptosis marker 4-HNE was also markedly elevated under both conditions, reflecting lipid peroxidation-induced oxidative stress (Fig. 6H, I). In addition, c-Fos expression increased following RSL3 treatment, whereas Fer-1 treatment reversed this effect (Fig. 6J, K). These findings indicate that GPX4-dependent ferroptosis in DRG neurons contributes to the onset and progression of neuropathic pain in CCI mice.

## Discussion

The regulatory mechanism of ferroptosis as a mode of neuronal death in neuropathic pain remains unclear [33]. In this study, PARP1-mediated ferroptosis in DRG neurons was identified as a key driver of neuropathic pain after nerve injury. Using transgenic mouse models, behavioral testing, and electrophysiology, we demonstrated that PARP1 downregulates GPX4 expression, thereby promoting DRG neuron ferroptosis and pain hypersensitivity.

Nerve injury and inflammation are considered key pathological mechanisms underlying neuropathic pain [5, 6]. PARP1, a critical DNA damage repair enzyme, has been implicated in neuropathic pain models [16]. Recent studies have confirmed that neurons ferroptosis contributes to neurodegenerative diseases and neuropathic pain [26, 27]. The study found that PARP1 expression and activity were significantly elevated in CCI mice. Conditional knockout of PARP1 in neurons mitigated CCI-induced neuropathic pain-like behavior and suppressed ferroptosis. Furthermore, PJ34 and olaparib inhibited PARP1 activity and attenuated nerve injury-induced pain sensitization while decreasing reducing Fe^2+^ overload, lipid oxidation, and ferroptosis in DRG neurons. Nerve injury causes abnormal action potentials in affected nerves, leading to hyperexcitability of DRG neurons [34, 35]. Consistently, the results showed that CCI significantly promoted DRG neuron excitability. This heightened excitability was reduced by PARP1 knockout in DRG neurons. Accordingly, these data suggest that overactivated PARP1 in DRG neurons is both necessary and sufficient for inducing neuronal hyperexcitability. Previous studies using different neuropathic pain models have revealed sex differences in spinal microglial transcriptomes [36], suggesting that gender may affect the manifestation of neuropathic pain and therapeutic responses. To evaluate PARP1 inhibiton across sexes, male and female CCI mice were treated with PJ34 or olaparib. PARP1 inhibition effectively reduced pain sensitization in both sexes, indicating that PARP1 contributes to neuropathic pain by mediating ferroptosis in DRG neurons.

Ferroptosis is a recently identified form of cell death marked by iron accumulation and lipid peroxidation [17], and growing evidence links it to neurodegenerative diseases and pain conditions [37, 38]. Iron homeostasis imbalance and lipid peroxidation can trigger ferroptosis through GPX4-dependent and non-GPX4-dependent pathways, as well as abnormal expression of iron transport proteins [39]. To clarify the mechanism by which PARP1 mediates DRG neuron ferroptosis in neuropathic pain, we analyzed the expression of ferroptosis-related genes in DRG neurons from the CCI model. GPX4 expression was downregulated in the CCI mice, whereas its expression significantly increased upon PARP1 inhibition. To determine whether GPX4-dependent ferroptosis contributes to neuropathic pain-like behavior, we treated normal and CCI mice with RSL3, a GPX4 inhibitor, and Ferrostatin-1, a ferroptosis blocker. GPX4 inhibition induced neuropathic pain-like behavior and DRG neuron ferroptosis in normal mice, while suppressing ferroptosis alleviated pain sensitization in CCI mice. These findings suggested that PARP1 drives DRG neuron ferroptosis by downregulating GPX4 expression in neuropathic pain.

GPX4 transcription is regulated by SP1, a DNA-binding transcription factor [40]. PARP1 modulates gene expression through poly(ADP-ribosyl)ation of transcription factors [41]. For instance, PARP1-mediated poly(ADP-ribosyl)ation of signal transducer and activator of transcription 3 suppresses programmed cell death ligand 1 transcription in cancer cells [42]. Consistent with this, poly(ADP-ribosyl)ation of SP1 was enhanced in DRG neurons of CCI mice. This study demonstrated that increased SP1 poly(ADP-ribosyl)ating in DRG of CCI mice, and that PARP1 knockout inhibited this modification. These findings suggest that PARP1 suppresses GPX4 expression by promoting SP1 poly(ADP-ribosyl)ation, thereby facilitating ferroptosis. It must be acknowledged that this study has a limitation. While our Co-IP results indicate PARP1–SP1 interaction, and previous studies has shown PARP1 mediates SP1 poly(ADP-ribosyl)ation, the direct causal role of SP1 PARylation in GPX4 downregulation within our CCI model remains inferred rather than experimentally validated.

## Conclusion

This study identifies PARP1-mediated, GPX4-dependent ferroptosis in DRG neurons as a critical mechanism underlying neuropathic pain. Following nerve injury, overactivated PARP1 downregulates GPX4 expression in these neurons. This leads to iron overload, lipid peroxidation, and ferroptosis, thereby driving the development of neuropathic pain. Pharmacological inhibition or conditional knockout of PARP1 effectively alleviated pain behaviors and suppressed ferroptosis.

This work identifies a regulatory mechanism of neuronal cell death in neuropathic pain, linking PARP1’s established role in DNA damage repair to the execution of ferroptosis. Targeting the PARP1-GPX4 axis through inhibition or genetic modulation may provide a viable therapeutic approach for managing neuropathic pain.

## Materials and methods

### Experimental animals

All animal experiments were approved by the Animal Ethics Committee of Zhujiang Hospital, Southern Medical University (Ethics No. LAEC-2022-024FS, Guangzhou, China) and performed in accordance with the relevant guidelines and regulations. The study was reported in compliance with the ARRIVE guidelines. Mice were housed in a specific pathogen-free environment (4–6 animals per cage) with unrestricted access to food and water. Wild-type C57BL/6N mice (4–6 weeks old, both sexes) were obtained from Viton Lihwa. The conditional PARP1 knockout mice (CKO, CKOAI191017MG1) on a C57BL/6N background were obtained from Cyagen Biosciences (Guangzhou, China). Mice were numbered and randomly assigned to four experimental groups using a computer-generated random number sequence. The cages were labeled only with numbers, and the investigators remained blinded to group allocation during data acquisition. Only mice that recovered uneventfully from surgery and exhibited substantial postoperative pain hypersensitivity were included in the subsequent analysis.

Neuronal PARP1 knockout mice (genotype *Nestin*^cre^*PARP1*^flox/flox^) and control mice (genotype *PARP1*^flox/flox^) were generated using CRISPR/Cas9 and Cre-loxP recombination. Guide ribonucleic acid (gRNA) targeting the PARP1 gene (NCBI-ID: 11545, Supplementary Table 1), a loxP vector, and Cas9 messenger RNA were used to produce F0 homozygous *PARP1*^flox/flox^ mice. These mice were backcrossed to obtain F1 and F2 generations. F2 homozygous mice were then crossed with heterozygous Nestin-Cre mice to obtain neuronal conditional knockdown offspring.

Dose-response experiments were conducted using PJ34 (5, 10, and 20 mg/kg) and olaparib (1, 5, and 10 mg/kg) to identify the optimal doses for subsequent experiments. Wild-type male mice were treated with PJ34 (Selleck S7300) and divided into four groups (*n* = 6 per group) : Sham + normal saline (NS; intraperitoneal injection of an equivalent dose of 0.9% normal saline), Sham + PJ34 (intraperitoneal injection of PJ34 at 10 mg/kg/day, diluted in 0.9% saline), CCI + NS, and CCI + PJ34. Wild-type female mice were treated with olaparib (AbMole M1664) and similarly divided into four groups (*n* = 6 per group): Sham + DMSO (intraperitoneal injection of an equivalent dose of DMSO), Sham + Olaparib (intraperitoneal injection of Olaparib at 5 mg/kg/day, diluted with DMSO), CCI + DMSO, and CCI + Olaparib.

Wild-type male mice treated with RSL3 (Selleck S8155) or Ferrostatin-1 (Fer-1, Selleck S7243) were divided into four groups (*n* = 6 per group): Sham + DMSO (intrathecal injection of an equivalent volume of DMSO), Sham + RSL3 (intrathecal injection of 20 μg RSL3 in 10 μL DMSO), CCI + DMSO, and CCI + Fer-1 (intrathecal injection of 20 μg Fer-1 in 10 μL DMSO).

### Pain model

The CCI model was established as previously described [43]. Mice were anesthetized and placed on a warming pad. The sciatic nerve was exposed, and three loose ligatures were placed around it with 5-0 silk thread until a slight muscle twitch appeared. Sham-operated mice underwent nerve exposure without ligation. Animals were allowed to recover for 48 h before behavioral testing.

### Behavioral tests

Mice were acclimated for 48 h before behavioral testing. Baseline values were recorded 24 h before surgery. Mechanical and thermal sensitivities were assessed on postoperative days 3, 7, 10, 14, 21, and 28. Before testing, mice were acclimated to the testing environment for 30 min to keep them in a calm state, with the upper limit for thermal radiation set at 20 s to prevent overheating injury. For the Von Frey test, mice were placed in opaque boxes on a wire mesh, and the mechanical paw withdrawal threshold was measured using Von Frey filaments of different forces according to the up-down method [44]. Thermal tests were conducted using the Hargreaves method [45], and the positive response was recorded as the average of three trials with intervals of at least 5 min.

### Western blotting

L_4_–L_6_ DRG tissues were lysed in buffer containing protease and phosphatase inhibitors at –20 °C. Protein concentrations were measured, denatured, and separated by SDS-PAGE. After transfer, membranes were blocked and incubated overnight at 4 °C with primary antibodies, then with secondary antibodies. Chemiluminescence signals were detected and analyzed using ImageJ. The major antibodies included PARP1 Antibody (Abmart T55347), Poly/Mono-ADP Ribose (E6F6A) Rabbit mAb (CST 83732S), GPX4 Antibody (Abmart T56959), Ferritin heavy chain Polyclonal Antibody (Immunoway YT1692), Sp1 Monoclonal Antibody (Genetex GTX636836), β-actin (Beijing Rui Kang RM2001), Goat anti-Rabbit IgG (H + L)-HRP (Beijing Rui Kang RM3002), and Goat anti-Mouse IgG (H + L)-HRP (Beijing Rui Kang RM3001).

### Real-time PCR

RNA was extracted with AG RNAex Pro (Eicole Bio AG21102), purified with chloroform, isopropanol, and 75% ethanol, and reverse transcribed with Evo M-MLV reagents (Eicole Bio AG11728) to generate cDNA. Target primers (Supplementary Table 2), sample cDNA, and SYBR Green Pro Taq HS pre-mixed qPCR reagents (Eicole Bio AG11701) were mixed in eight-tube strips. Real-time fluorescence quantitative amplification was performed on a Roche LightCycle 480 II, and data were analyzed using the 2^–ΔΔCt^ method.

### Immunofluorescent staining

Immunofluorescence was performed using a dual-marker three-color fluorescence detection kit for mouse and rabbit antibodies (Immunoway RS0036). After fixation, permeabilization, and blocking, tissues were incubated with primary antibodies at 4 °C, followed by secondary antibodies, TSA dyes (D-594, D-488), and DAPI counterstaining. Confocal imaging was performed with a Nikon microscope, and images were analyzed with ImageJ. The main antibodies employed in the study include PARP1 (Abmart T55347), Ferritin Heavy (Immunoway YT1692), Goat anti-Rabbit IgG (H + L)-HRP (Ray Antibody RM3002), c-Fos (9F6) Rabbit mAb (CST 2250 T), Anti-4 Hydroxynonenal antibody (Bioss bs-6313R), and NeuN Antibody (Abmart T55515).

### Intracellular Fe^2+^ detection

DRG neurons were isolated from the L_4_–L_6_ regions, enzymatically digested with Collagenase type I (Gibco 17100017) and Dispase type II (Sigma-Aldrich D4693), and cultured in Neurobasal-A medium (Gibco 10888022) with B27 supplement (Gibco 17504044) and Glutamax (Invitrogen 35050). FerroOrange working solution (DOJINDO F374, 1:1000) was added and incubated for 30 min, followed by DAPI staining (Absin abs9235). Observations and imaging were performed using a Nikon confocal microscope, and analysis was carried out with ImageJ.

### Co-immunoprecipitation

L_4_–L_6_ DRG tissues were isolating from mice, followed by the addition of lysis buffer and protease inhibitors and protein extraction using a grinder. The protein concentration of input samples was determined, and the samples were heat-denatured and stored until use. The immunoprecipitation (IP) group received target protein antibodies, while the IgG group received IgG antibodies, and both were incubated on a rotating shaker at 4 °C overnight. Magnetic beads were added the next day to both groups to pellet immunocomplexes, with incubation continuing at 4 °C overnight. On the third day, the beads were washed, resuspended in SDS sample buffer, and heat-denatured. Primary antibodies include Sp1 (Abmart PA3320S), and Mouse Anti-Rabbit IgG HRP (Abmart M21006S) was used as the secondary antibody.

### Gene identification

Genotyping was performed based on the manufacturer’s protocol (BioYunTian D7283). Mouse tail samples were processed with DNA Extraction Solution and Enzyme Mix to obtain genomic DNA. PCR Master Mix, primers (Supplementary Table 1), and DEPC water were combined and amplified in a PCR machine. PCR products were separated by agarose gel electrophoresis and documented using a gel imaging system.

### Patch clamp

Patch-clamp recordings were performed as previously described [34], with minor modifications. Mice were deeply anesthetized and transcardially perfused with oxygenated (95% O_2_/5% CO_2_) ice-cold N-methyl-D-glucamine (NMDG) cutting solution (Brainvta) comprising (in mM): 93 NMDG, 93 HCl, 2.5 KCl, 1.2 NaH_2_PO_4_, 30 NaHCO_3_, 25 D-glucose, 20 HEPES, 5 Na-ascorbate, 2 thiourea, 3 Na-pyruvate, 10 MgSO_4_, and 0.5 CaCl_2_ (pH 7.35). DRG tissue was isolated and sliced at 250 μm thickness using a Leica VT1200s vibratome, then maintained at 25 °C in oxygenated artificial cerebrospinal fluid (ACSF; in mM: 126 NaCl, 2.5 KCl, 0.5 CaCl_2_, 2 MgCl_2_, 26 NaHCO_3_, 1.25 NaH_2_PO_4_,10 glucose) before recording. For whole-cell recording, pipettes were filled with a solution containing (in mM): 135 K-gluconate, 5 KCl, 0.5 CaCl_2_, 10 HEPES, 2 Mg-ATP, 0.1 GTP, and 5 EGTA (300 mOsm, pH adjusted to 7.3 with KOH). Action potentials (APs) were elicited by 400-ms current injections at 10 intensities (0–90 pA, 10 pA increments), with a 20-s inter-stimulus interval. The frequency of AP was defined as the number of spikes per time window. All signals were acquired with a MultiClamp 700B amplifier (Molecular Devices).

### Statistical analysis

Data were tested for normality using the Shapiro–Wilk test. Homogeneity of variance was assessed using Levene’s test. Comparisons between two groups were made using unpaired two-tailed Student’s *t* test. For comparisons across different groups and treatment conditions, two-way analysis of variance (ANOVA) was performed, followed by Tukey’s post hoc test for multiple comparisons. Non-normally distributed data were analyzed using the Kruskal–Wallis test with Dunn’s post hoc procedure.Data are presented as mean ± standard error of the mean. A *P* value < 0.05 was considered statistically significant. All statistical analysis was conducted using ImageJ and GraphPad Prism 9.

Sample size was determined using G*Power 3.1 software. The experimental design employed a two-way mixed repeated measures ANOVA, with modeling (Sham/CCI) and treatment (vehicle/PARP inhibitor) as between‑subjects factors and time as the within‑subjects factor. With α = 0.05, power = 0.80, and a correlation among repeated measures set at r = 0.5, the calculation indicated that 6 mice per group were required. Accordingly, a total of 24 mice (4 groups × 6 mice) were included in this study.

## Supplementary information

Supplementary Figure Legend

Supplementary Figure

Supplementary Table

Western blots

## Data Availability

All data are available from the corresponding author upon reason able request.
