# Supplementary material for: PARP1 contributes to neuropathic pain via GPX4-dependent sensory neuron ferroptosis in nerve-injured mice
Source: Cell Death Discov. 2026 Aug 15;12:454. doi: 10.1038/s41420-026-03307-4 (PMC13755442; doi:10.1038/s41420-026-03307-4)
Supplement: Supplementary file 1 [file 41420_2026_3307_MOESM1_ESM.docx]

**Supplementary Fig.1 PARP1 expression in various tissues of PARP1 conditional knockout mice and genotyping by nucleic acid electrophoresis.**

(A) PCR nucleic acid electrophoresis shows that the amplicon from wild-type mice has a smaller base pair (bp) size compared to that from *PARP1*^NES/NES^ mice.

(B-E) Verification of PARP1 knockdown in the DRG (B), heart (C), liver (D), and lung (E) was performed by qPCR and Western blot analyses following conditional PARP1 knockout.

Data are expressed as mean ± SEM (n = 3).
