# Supplementary material for: PARP1 contributes to neuropathic pain via GPX4-dependent sensory neuron ferroptosis in nerve-injured mice
Source: Cell Death Discov. 2026 Aug 15;12:454. doi: 10.1038/s41420-026-03307-4 (PMC13755442; doi:10.1038/s41420-026-03307-4)
Supplement: Supplementary file 2 [file 41420_2026_3307_MOESM3_ESM.docx]

**Supplementary Table 1 gRNA targeting the PARP1 gene**

| gRNA | target sequence (matching reverse strand) |
| --- | --- |
| gDNA1 | GGCCACTTTTTTCGCCTATGAGG |
| gDNA2 | CTAGGTTCTATCAGACGCACTGG |

**Supplementary Table 2 Detection of Ferroptosis-Related Gene Sequences Using RT-qPCR**

| Gene | Primer（5’→3’） |
| --- | --- |
| PARP1 | Forward: GGCAGCCTGATGTTGAGGT |
|  | Reverse: CGTACTCCGCTAAAAAGTCAC |
| FSP1 | Forward: CGGGTTCGCCAAAAAGACATT |
|  | Reverse: CTATGCCAATCACTTTGCCCT |
| TFR1 | Forward: GTTTCTGCCAGCCCCTTATTAT |
|  | Reverse: GCAAGGAAAGGATATGCAGCA |
| DMT1 | Forward: TACCTAGACCCAGGAAACATCG |
|  | Reverse: CACTCCAAGTCTCGCTGCAA |
| GPX4 | Forward: GATGGAGCCCATTCCTGAACC |
|  | Reverse: CCCTGTACTTATCCAGGCAGA |
| FPN1 | Forward: ACCAAGGCAAGAGATCAAACC |
|  | Reverse: AGACACTGCAAAGTGCCACAT |
| β-actin | Forward: GGCTGTATTCCCCTCCATCG |
|  | Reverse: CCAGTTGGTAACAATGCCATGT |
