# Supplementary material for: PARP1 contributes to neuropathic pain via GPX4-dependent sensory neuron ferroptosis in nerve-injured mice
Source: Cell Death Discov. 2026 Aug 15;12:454. doi: 10.1038/s41420-026-03307-4 (PMC13755442; doi:10.1038/s41420-026-03307-4)
Supplement: Supplementary file 3 [file 41420_2026_3307_MOESM4_ESM.docx]

Figure2A - β-actin

β-actin

Figure2A - PARP1

PARP1

Figure2B - PAR

PAR

Figure2B - β-actin

β-actin

Figure5C - GPX4 & β-actin

GPX4

β-actin

Figure5D - PARP1

PARP1

Figure5D - β-actin

β-actin

β-actin

Figure5E - PAR & β-actin

PAR

β-actin

Figure5I - SP1

SP1

Figure5I – β-actin

β-actin

Figure5J - SP1

SP1

Figure5J – β-actin

β-actin

Figure5K - PARP1（CO-IP）

PARP1

Figure5K - SP1（CO-IP）

SP1

Figure5L - PAR（CO-IP）

PAR

Figure5L - SP1（CO-IP）

SP1
